# Advancing Physical Literacy Research in Children

**DOI:** 10.3390/children11060702

**Published:** 2024-06-07

**Authors:** Elizabeth J. Durden-Myers

**Affiliations:** 1 School of Education, Bath Spa University, Bath BA1 5SF, UK; e.durden-myers@bathspa.ac.uk; 2 School of Education and Science, The University of Gloucestershire, Cheltenham GL50 4AZ, UK

**Keywords:** physical literacy, children, youth, physical education, disability, indigenous, parents, assessment, health and wellbeing

## Abstract

The collection of papers in this Special Issue serves to extend the literature and evidence base for physical literacy (PL) research within child and youth populations. Currently, child and youth populations are increasingly sedentary, resulting in them spending less time engaging in daily physical activity (PA). Physical literacy serves as an attractive concept to help reframe and address physical inactivity and poor health and wellbeing, utilising a different and integrated approach to physical activity, health and wellbeing promotion. The studies presented in this Special Issue respond to previous calls in PL research for further empirical evidence, clarity around PL assessment, the utility of physical literacy with diverse populations including indigenous children and those with disabilities, the application of PL within early years, parental engagement and the role of physical education in the promotion of PL. These studies shed new light on the frontiers of PL research within child and youth populations.

## 1. Introduction

In recent years, the concept of physical literacy has gained prominence globally [1]. Physical literacy (PL) has applications in various sectors such as sport, health, education and recreation and throughout the life course [2,3]. This Special Issue is dedicated to the exploration of PL, with a particular emphasis on physical literacy development for children and youths. The goal is to disseminate leading research and practical insights from diverse contexts worldwide, shedding light on the foundational aspects that contribute to a physically active and flourishing life. The studies presented in this Special Issue respond to previous calls in the field of PL for the following research:

- Further empirical evidence making the link between PL and improved health and wellbeing outcomes, and evidence specifically within child and youth populations [4,5].
- Clearer methods for charting progress and assessment that are aligned to the philosophical underpinning of the concept [4,6].
- The utility of PL with diverse populations, including indigenous children and those with disabilities [7].
- The application of PL within early years and parental engagement [8].
- The role of PL in the physical education environment [9,10,11].

The collection of papers in this Special Issue serves to extend the literature and evidence base for PL research within child and youth populations. 

Child and youth populations are increasingly sedentary, resulting in them spending less time engaging in daily physical activity (PA) [12]. It is estimated that four out of five adolescents aged 13–15 years do not meet the current minimum recommendations for daily PA [12]. Considering the World Health Organization’s (WHO) recommendations regarding PA, young people should engage in 60 min of moderate to vigorous PA per day, yet more than 80% of adolescents, mostly in EU member states, do not meet these recommendations [13]. Concurrently, mental health issues have been significantly exacerbated by the COVID-19 pandemic [14] and are increasing. To address and reverse these trends, the United Nations Educational, Scientific and Cultural Organization (UNESCO) [15] is calling for nations to develop inclusive sport policies that focus on the delivery of evidence-based sport and quality PE interventions in schools and communities. Therefore, it is understandable that PL serves as an attractive concept that uses a different and integrated approach to help reframe and address physical inactivity and poor health and wellbeing. The studies included in this Special Issue shed new light on the frontiers of PL research within child and youth populations.

## 2. Role of Satisfaction in Life, Sex and Body Mass Index in Physical Literacy of Spanish Children

The first study in this Special Issue by Urbano-Mairena et al. [16] investigated the relationship between physical literacy (PL), life satisfaction (LS) and body mass index (BMI) in children aged 8–12 years. PL, crucial for good health and preventing cardiovascular diseases and obesity, was found to be lower in children who were categorised as overweight or obese. PL positively correlated with LS but inversely correlated with BMI. Daily physical activity (PA) behaviour was influenced by LS and sex, while physical competence was influenced by LS and BMI. Motivation and confidence were only associated with LS. The study concluded that children with a BMI in the acceptable range had higher PL and LS levels.

The empirical evidence base in support of the relationship between physical literacy, engagement in PA and LS is increasing. Empirical research is uncovering the link between PL, PA and health and wellbeing outcomes [5]. This increasing empirical evidence is continuing to validate the importance of PL as a tool for understanding engagement in PA and supporting health and wellbeing outcomes. With the growing evidence base, the momentum surrounding PL research and implementation across multiple sectors seems unlikely to slow. 

## 3. Evaluation of Physical Literacy in Southeastern European Countries

The second study in the Special Issue by Vuletic et al. [17] conducted research in three Southeastern European countries (Croatia, Bosnia and Herzegovina, and Montenegro), and delved into the reliability and validity of measurement tools for assessing physical literacy in 9-to-11-year-old children. The research highlights the significance of context-specific evaluation tools, demonstrating that the PLAYself questionnaire surpasses the CAPL-2 questionnaire in reliability. Moreover, the study establishes a positive association between physical literacy and sport participation, validating the efficacy of the PLAYself questionnaire.

The literature base associated with physical literacy assessment that accounts for validity, feasibility and fidelity is becoming increasingly sophisticated. Yet a debate remains regarding whether physical literacy can even be measured. Therein lies the tension between ideological and real-world interpretations of physical literacy [18]. This will continue to be a well-contested area within future research. 

## 4. The Meaning of Physical Literacy for Instructors of Children with Disabilities

The third study in the series by Pushkarenko, Causgrove Dunn and Goodwin [19] uses interpretive phenomenological analysis to explore the perspectives of instructors engaged in facilitating physical activity for children with disabilities. Guided by ecological systems theory, the study identified four key themes: recognising unique embodiments, emphasising the importance of context, extending beyond physical competence, and navigating the dominant discourse surrounding physical literacy. Instructors emphasise the crucial role of movement skill development while embracing diversity, exploratory play, partial participation, family involvement, and flexibility in pedagogy.

Physical literacy impresses the importance of inclusion and celebrates the unique physical literacy journal of all individuals [3]. Yet scant research exists across a range of diverse populations. This research is much needed and welcomed in the field, and it is encouraging to see this gap in the literature being addressed. 

## 5. Piloting the Virtual PLAYshop Program

The fourth study in the Special Issue by Hwang et al. [20] outlines a single-group, mixed-methods pilot study to evaluate the feasibility and outcomes of the Virtual PLAYshop program, a parent-focused physical literacy intervention for early childhood. The virtual delivery, including workshops, resources and booster emails, was demonstrated to result in high satisfaction rates among parents. The assessment protocol for children’s fundamental movement skills proved feasible and yielded positive changes, supporting the potential of virtual interventions in promoting physical literacy. The study recommends further exploration through a larger randomised, controlled efficacy trial.

The preschool years are a critical period of physical literacy development. Previous evidence has shown that meaningful parental involvement plays a crucial role in shaping physical activity-related behaviours in children [21,22]. Relatively little is understood about how best to support parents in nurturing their children’s physical literacy. This pilot study began to explore this gap in the literature.

## 6. Bibliometric Analysis of Physical Literacy Studies

The fifth study in the series is a comprehensive bibliometric analysis by Urbano-Mairena et al. [23] and provides a globalised view of physical literacy studies related to the health of children and adolescents. Spanning 141 documents from 2014 to 2022, the analysis reveals an exponential growth in research, with contributions from 37 countries and regions. The identification of prolific authors, journals and keywords enhances our understanding of the evolving landscape of physical literacy and highlights the variety of emerging research in the field across the globe. 

## 7. Nature’s Way–Our Way Pilot Project Case Assemblage

The penultimate paper in the Special Issue is a case assemblage by Riley et al. [24] that employs a post-qualitative approach and new materialist methodology to explore the Nature’s Way–Our Way (NWOW) initiative. Focused on indigenous early-childhood education in Saskatchewan, Canada, the study highlights how the NWOW initiative negotiates movement with early-childhood educators. It emphasises the role of land in shaping physical literacy stories, serving as a vital protective factor for indigenous preschool-aged children’s holistic wellness.

This paper unravels several unknowns within physical literacy research. New materialism and post-qualitative methodologies offer a novel way to explore physical literacy through philosophically aligned paradigms. The pairing of appropriate methods with contextual environments serves as a useful reminder to select sensitive methodologies to support the exploration of symbiotic relationships and ideologies between cultures. 

## 8. Physical-Literacy-Enriched Physical Education: A Capabilities Perspective

The final article by Durden-Myers and Bartle [25] adopts a post-qualitative sensibility to understand the value of physical literacy as the goal of physical education through the lens of the capability approach. Aligning physical literacy with the ten capabilities proposed by Nussbaum [26], the paper advocates for physical-literacy-enriched physical education as a foundation for holistic development and lifelong engagement in physical activity. The discussion extends from traditional humanist perspectives to post-humanism, offering a more holistic and ecological appreciation of the relationship between capabilities, physical literacy, and physical education.

This paper is an example of how the philosophical foundations of physical literacy are continuing to grow and expand into wider areas, promoting further philosophical discussions. Considering that the origins of physical literacy are grounded in philosophical exploration [27], it is fantastic to see the philosophical grounding continuing over 20 years later. 

## 9. Conclusions

Collectively, this Special Issue offers a rich tapestry of insights into advancing physical literacy in children and youths. From evaluating measurement tools and exploring cultural contexts to promoting virtual interventions, the contributions underscore the multidimensional nature of physical literacy. These studies emphasise the importance of physical literacy as a framework to support children’s wellbeing, development and lifelong engagement in physical activity, urging further exploration and integration of physical literacy within educational and children and youth policy frameworks globally.

## References

[1] Shearer C., Goss H., Edwards L.C., Keegan R.J., Knowles Z.R., Boddy L.M., Durden-Myers E.J., Foweather L. (2018). How is Physical Literacy Defined? A Contemporary Update. J. Teach. Phys. Educ..

[2] Durden-Myers E.J., Meloche E.S., Dhillon K.K. (2020). The Embodied Nature of Physical Literacy: Interconnectedness of Lived Experience and Meaning. J. Phys. Educ. Recreat. Danc..

[3] Whitehead M. (2010). Physical Literacy throughout the Lifecourse.

[4] Green N.R., Roberts W.M., Sheehan D., Keegan R.J. (2018). Charting Physical Literacy Journeys Within Physical Education Settings. J. Teach. Phys. Educ..

[5] Melby P.S., Nielsen G., Brønd J.C., Tremblay M.S., Bentsen P., Elsborg P. (2022). Associations between children’s physical literacy and well-being: Is physical activity a mediator?. BMC Public Health.

[6] Edwards L.C., Bryant A.S., Keegan R.J., Morgan K., Cooper S.M., Jones A.M. (2018). Measuring’ Physical Literacy and Related Constructs: A Systematic Review of Empirical Findings. Sports Med..

[7] Pushkarenko K., Causgrove Dunn J., Wohlers B. (2021). Physical literacy and inclusion: A scoping review of the physical literacy literature inclusive of individuals experiencing disability. Prospects.

[8] Carl J., Barratt J., Töpfer C., Cairney J., Pfeifer K. (2022). How are physical literacy interventions conceptualized?—A systematic review on intervention design and content. Psychol. Sport Exerc..

[9] Durden-Myers E.J. (2020). Operationalising Physical Literacy within Physical Education Teaching Practice through Professional Development. Ph.D. Thesis.

[10] Houser N., Kriellaars D. (2023). “Where was this when I was in Physical Education” Physical Literacy Enriched Pedagogy in a Quality Physical Education Context. Front. Sport Act. Living.

[11] Stoddart A.L., Humbert M.L., Kerpan S., Cameron N., Kriellaars D. (2023). PLitPE: An intervention for physical literacy enriched pedagogy in Canadian elementary school physical education classes. Phys. Educ. Sport Pedagog..

[12] Sahoo K., Sahoo B., Choudhury A.K., Sofi N.Y., Kumar R., Bhadoria A.S. (2015). Childhood obesity: Causes and consequences. J. Fam. Med. Prim. Care.

[13] World Health Organisation (WHO) Global Status Report on Physical Activity 2022: Let’s Get Moving!. https://www.who.int/teams/health-promotion/physical-activity/global-status-report-on-physical-activity-2022.

[14] World Health Organisation (WHO) Young People Leading the Way to a Brighter Post-COVID World. https://www.who.int/news-room/feature-stories/detail/young-people-leading-the-way-to-a-brighter-post-covid-world.

[15] United Nations Educational, Scientific and Cultural Organization (UNESCO) Fit for Life: Sport Powering Inclusive, Peaceful and Resilient Societies. https://unesdoc.unesco.org/ark:/48223/pf0000379910.

[16] Urbano-Mairena J., Mendoza-Muñoz M., Carlos-Vivas J., Pastor-Cisneros R., Castillo-Paredes A., Rodal M., Muñoz-Bermejo L. (2024). Role of Satisfaction with Life, Sex and Body Mass Index in Physical Literacy of Spanish Children. Children.

[17] Vuletic P.R., Kesic M.G., Gilic B., Pehar M., Uzicanin E., Idrizovic K., Sekulic D. (2023). Evaluation of Physical Literacy in 9- to 11-Year-Old Children: Reliability and Validity of Two Measurement Tools in Three Southeastern European Countries. Children.

[18] Quennerstedt M., McCuaig L., Mårdh A. (2021). The fantasmatic logics of physical literacy. Sport Educ. Soc..

[19] Pushkarenko K., Causgrove Dunn J., Goodwin D. (2023). The Meaning of Physical Literacy for Instructors of Children Experiencing Disability, from an Ecological Systems Perspective. Children.

[20] Hwang Y., Boyd M., Naylor P.-J., Rhodes R.E., Liu S., Moldenhauer R., Li J., Wright C., Buckler E.J., Carson V. (2023). Piloting the Virtual PLAYshop Program: A Parent-Focused Physical Literacy Intervention for Early Childhood. Children.

[21] Messing S., Rütten A., Abu-Omar K., Ungerer-Röhrich U., Goodwin L., Burlacu I., Gediga G. (2019). How Can Physical Activity Be Promoted Among Children and Adolescents? A Systematic Review of Reviews Across Settings. Front. Public Health.

[22] Rhodes R.E., Guerrero M.D., Vanderloo L.M., Barbeau K., Birken C.S., Chaput J.P., Faulkner G., Janssen I., Madigan S., Mâsse L.C. (2020). Development of a consensus statement on the role of the family in the physical activity, sedentary, and sleep behaviours of children and youth. Int. J. Behav. Nutr. Phys. Act..

[23] Urbano-Mairena J., Castillo-Paredes A., Muñoz-Bermejo L., Denche-Zamorano Á., Rojo-Ramos J., Pastor-Cisneros R., Mendoza-Muñoz M. (2023). A Bibliometric Analysis of Physical Literacy Studies in Relation to Health of Children and Adolescents. Children.

[24] Riley K., Froehlich Chow A., Wahpepah K., Houser N., Brussoni M., Stevenson E., Erlandson M.C., Humbert M.L. (2023). A Nature’s Way—Our Way Pilot Project Case Assemblage: (Re)Storying Child/Physical Literacy/Land Relationships for Indigenous Preschool-Aged Children’s Wholistic Wellness. Children.

[25] Durden-Myers E.J., Bartle G. (2023). Physical-Literacy-Enriched Physical Education: A Capabilities Perspective. Children.

[26] Nussbaum M., de Greiff P., Cronin C.P. (2002). Capabilities and Human Rights. Global Justice and Transnational Politics.

[27] Whitehead M.E. (2001). The Concept of Physical Literacy. Eur. J. Phys. Educ..

